# Dissection and fine-mapping of two QTL for grain size linked in a 460-kb region on chromosome 1 of rice

**DOI:** 10.1186/s12284-018-0236-z

**Published:** 2018-08-02

**Authors:** Qing Dong, Zhen-Hua Zhang, Lin-Lin Wang, Yu-Jun Zhu, Ye-Yang Fan, Tong-Min Mou, Liang-Yong Ma, Jie-Yun Zhuang

**Affiliations:** 1 State Key Laboratory of Rice Biology and Chinese National Center for Rice Improvement, China National Rice Research Institute, Hangzhou, 310006 China; 2 State Key Laboratory of Crop Genetic Improvement and National Center of Plant Gene Research (Wuhan), Huazhong Agricultural University, Wuhan, 430070 China

**Keywords:** Close linkage, Grain size, Minor effect, Quantitative trait locus, Rice

## Abstract

**Background:**

Grain size is a key determinant of grain weight and a trait having critical influence on grain quality in rice. While increasing evidences are shown for the importance of minor-effect QTL in controlling complex traits, the attention has not been given to grain size until recently. In previous studies, five QTL having small effects for grain size were resolved on the long arm of chromosome 1 using populations derived from *indica* rice cross Zhenshan 97///Zhenshan 97//Zhenshan 97/Milyang 46. One of them, *qTGW1.2c* that was located in a 2.1-Mb region, was targeted for fine-mapping in the present study.

**Results:**

Firstly, the *qTGW1.2c* region was narrowed down into 1.1 Mb by determining genotypes of the cross-over regions using polymorphic markers newly developed. Then, one BC_2_F_9_ plant that was only heterozygous in the updated QTL region was identified. A total of 12 populations in generations from BC_2_F_11:12_ to BC_2_F_15:16_ were derived and used for QTL mapping. Two QTL linked in a 460-kb region were separated. The *qGS1-35.2* was delimited into a 57.7-kb region, containing six annotated genes of which five showed nucleotide polymorphisms between the two parental lines. Quantitative real-time PCR detected expression differences between near isogenic lines for *qGS1-35.2* at three of the six annotated genes. This QTL affected grain length and width with opposite allelic directions, exhibiting significant effect on ratio of grain length to width but showing little influence on yield traits. The other QTL, *qGW1-35.5*, was located within a 125.5-kb region and found to primarily control grain width and consequently affect grain weight.

**Conclusions:**

Our work lays a foundation for cloning of two minor QTL for grain size that have potential application in rice breeding. The *qGS1-35.2* could be used to modify grain appearance quality without yield penalty because it affects grain shape but hardly influences grain yield, while *qGW1-35.5* offers a new gene recourse for enhancing grain yield since it contributes to grain size and grain weight simultaneously.

**Electronic supplementary material:**

The online version of this article (10.1186/s12284-018-0236-z) contains supplementary material, which is available to authorized users.

## Background

Rice (*Oryza sativa* L.) is the staple food for more than half of the global population. Grain yield of rice depends on three components, i.e., number of panicles per plant, number of grains per panicle, and grain weight. Among them grain weight is mainly determined by grain size. Grain size and shape is also an important quality trait that greatly influences the market value of grain products. In general, a short and bold rice grain is favored by consumers in Northern China, Japan and Korea, while a long and slender rice grain is preferred by consumers in the Africa, America and countries of Southeast Asia (Calingacion et al., 2014).

Grain size and shape are largely determined by grain length and width. All of them are complex traits controlled by a large number of quantitative trait loci (QTL). Up to date, a total of 14 QTL having large effect for grain length and width in rice were cloned. One of them, *GL7*/*GW7*, has similar effects on grain length and width with opposite allelic directions, controlling grain shape but hardly influencing grain weight (Wang et al., 2015a; Wang et al., 2015b). The other 13 genes affect grain size and weight. Four of them mainly control grain width, including *GW2*, *GS5*, *qSW5/GW5*, and *GW8* (Li & Li, 2016). Eight others mainly control grain length, including *GS2*/*GL2*, *OsLG3*, *qLGY3*/*OsLG3b*, *GS3*, *GL3.1*/*qGL3*, *GL4*, *TGW6*, and *GLW7* (Li & Li, 2016; Wu et al., 2017; Yu et al., 2017; Liu et al., 2018; Yu et al., 2018). The remaining one, *GW6a*, has similar effects on grain length and width with the same allelic direction, and consequently exhibits a larger impact on grain weight (Song et al., 2015). It has been shown that these QTL regulate the proliferation and expansion of cells in spikelet hulls through diversified regulatory pathways. While most of them were involved in independent signaling pathways mediated by proteasomal degradation, plant hormones and G proteins, a number of genes were found to interact with each other (Yan et al., 2011; Wang et al., 2015a; Liu et al., 2018). These findings have greatly enriched our knowledge on the genetic control of grain size in rice, but much more efforts are needed to fill the gap in understanding the regulatory framework for this critical agronomical trait (Zuo & Li, 2014; Li & Li, 2016).

It has long been recognized that both major- and minor-effect QTL play important roles in the genetic control of complex traits (Mackay, Stone & Ayroles, 2009). QTL cloning in rice has been focused on those having large effects since the first success that was published in 2000 for heading date gene *Hd1* (Yano et al., 2000). Nevertheless, more and more attentions have been given to QTL with relative small effects in recent years. For heading date that has been taking the leading position in rice QTL studies, a number of minor-effect QTL were cloned or fine-mapped (Wu et al., 2013; Chen et al., 2014; Zhong et al., 2014; Chen et al., 2015; Shibaya et al., 2016). These QTL also showed important influences on the eco-geographical adaption and grain yield of rice, providing evidences for the importance of minor-effect QTL in controlling complex traits. For grain size and weight, QTL cloned are small in number and include no minor-effect QTL. While at least 546 QTL were detected in primary mapping and distributed over all regions of the 12 rice chromosomes (http://www.gramene.org), those that were cloned have very low genome coverage. None was located on chromosomes 1, 9, 10, 11 and 12, and on the long arm of chromosome 5 and short arms of chromosomes 2, 4, 6, 7 and 8. Isolation of QTL in these regions will be of great importance for establishing a gene network regulating grain size in rice.

In our previous studies, dissection of minor-effect QTL for grain weight and size was conducted using near isogenic lines (NILs) derived from a cross between *indica* rice cultivars Zhenshan 97 (ZS97) and Milyang 46 (MY46). Five QTL were resolved in an 8.2-Mb region on the long arm of chromosome 1 (Zhang et al., 2016). The present study aimed to fine-map one of the QTL, *qTGW1.2c* that was located in a 2.1-Mb interval (Wang et al., 2015c). Two linked QTL were separated in the target region, designated as *qGS1-35.2* and *qGW1-35.5*, respectively. The *qGS1-35.2* was delimited into a 57.7-kb region starting from the position of 35.2 Mb, affecting grain length and width with opposite allelic directions and showing little influence on grain weight. The *qGW1-35.5* was mapped in a 125.5-kb region starting from the position of 35.5 Mb, mainly controlling grain width and consequently affecting grain weight.

## Methods

### Plant materials

**Fig. 1:**
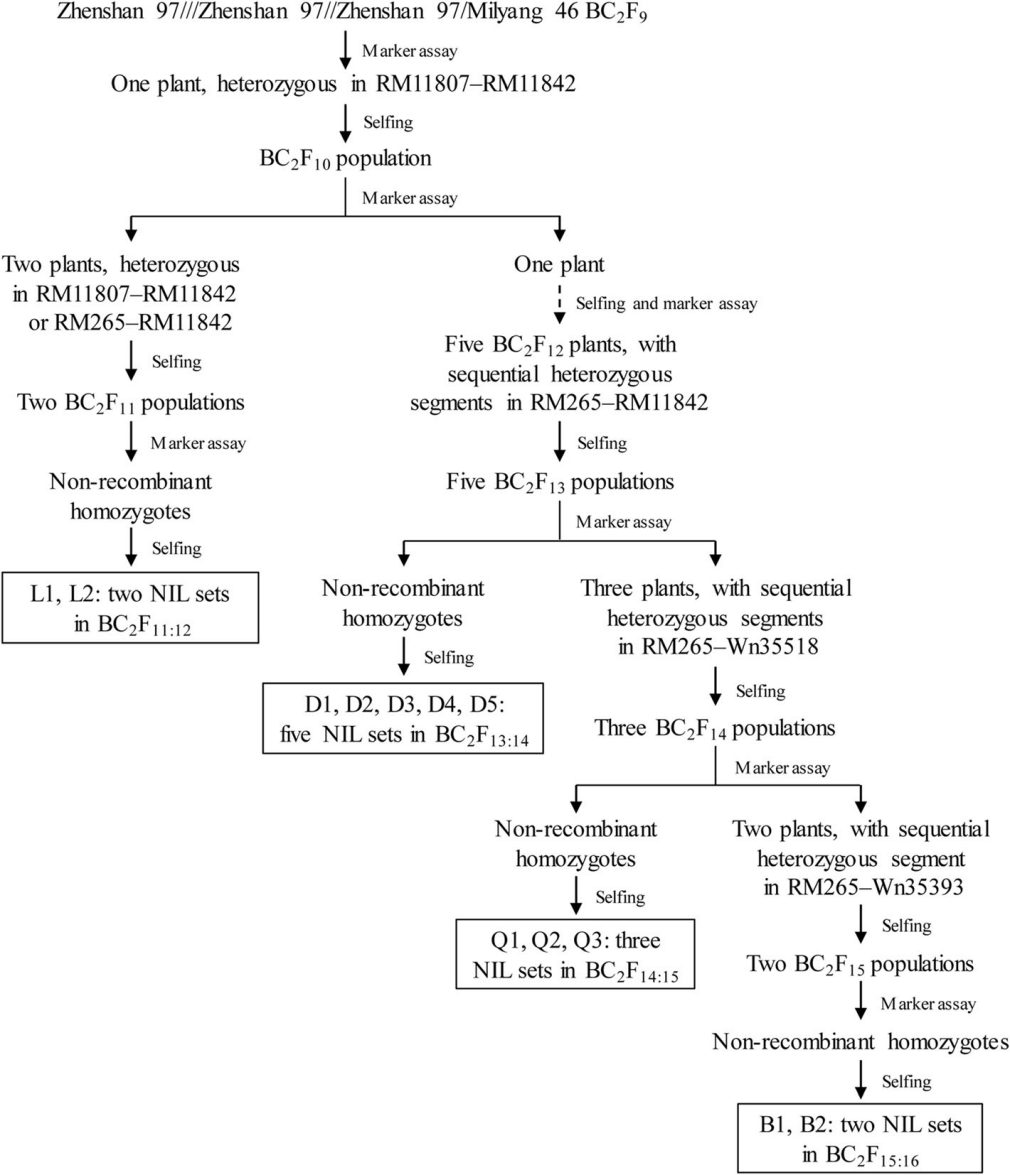
Development of the rice populations used in this study

A total of 12 populations segregating in an isogenic background were used in this study. As described below and illustrated in Fig. 1, they were derived from a BC_2_F_9_ plant of the rice cross ZS97///ZS97//ZS97/MY46.

Firstly, new polymorphic markers were developed in the cross-over regions of *qTGW1.2c* (Wang et al., 2015c) and used to determine genotypes of a set of NILs that segregated this QTL. The *qTGW1.2c* region was narrowed down to be RM11807–RM11842 (details are presented in the first section of Results). Then, a BC_2_F_9_ plant that was only heterozygous in the RM11807–RM11842 interval was identified. The resultant BC_2_F_10_ population consisting of 293 plants was genotyped using polymorphic markers in the target region. Two plants were selected, carrying heterozygous segments RM11807–RM11842 and RM265–RM11842, respectively. In the two resultant BC_2_F_11_ populations consisting of 246 and 111 plants, respectively, homozygous non-recombinants (i.e., plants that were homozygous and showed no recombination in the corresponding segregating region) were identified and selfed. Two sets of NILs, namely L1 and L2, were developed and used for QTL analysis. The *qTGW1.2c* region was updated to be RM265–RM11842.

Another BC_2_F_10_ plant carrying the RM265–RM11842 heterozygous segment was selected and selfed for two generations. A BC_2_F_12_ population consisting of 259 individuals was genotyped. Five plants were selected, carrying sequential heterozygous segments extending from RM265 to RM11842. In the five resultant BC_2_F_13_ populations consisting of 184, 188, 212, 190 and 206 plants, respectively, homozygous non-recombinants were identified and selfed. Five sets of NILs, namely D1, D2, D3, D4 and D5, were developed and used for QTL analysis. Two QTL were resolved, of which *qGS1-35.2* located in the upstream region was selected for further analysis.

Three plants were selected from the BC_2_F_13_ populations, carrying sequential heterozygous segments covering *qGS1-35.2*. In the three resultant BC_2_F_14_ populations consisting of 175, 166 and 187 plants, respectively, homozygous non-recombinants were identified and selfed. Three sets of NILs, namely Q1, Q2 and Q3, were developed and used for QTL analysis. The segregating region for *qGS1-35.2* was updated to be RM265–Wn35263.

Two other plants were selected from the BC_2_F_14_ populations, carrying heterozygous segments RM265–Wn35263 and RM11824–Wn35393, respectively. In the two resultant BC_2_F_15_ populations consisting of 199 and 237 plants, respectively, homozygous non-recombinants were identified and selfed. Two sets of NILs, namely B1 and B2, were developed and used for fine-mapping of *qGS1-35.2*.

### Field experiments and phenotyping

The rice populations were tested in the experimental stations of the China National Rice Research Institute located at either Hangzhou in Zhejiang Province or Lingshui in Hainan (Table 1). The experiments followed a randomized complete block design with two replications. In each replication, one line was grown in a single row of ten plants. Seedlings of about 25-day-old were transplanted with a planting density of 16.7 cm × 26.7 cm. Field management followed the normal agricultural practice. At maturity, five middle plants in each row were harvested in bulk. Fully filled grain were selected and evaluated for grain weight and size following the procedure reported by Zhang et al. (2016). Four traits were measured for all populations, including 1000-grain weight (TGW, g), grain length (GL, mm), grain width (GW, mm) and ratio of grain length to width (RLW). One more trait for grain size, grain thickness (GT, mm), was measured for populations B1 and B2. The measurement was made over 20 fully filled grains using an electronic digital caliper with a precision of 0.001 mm (Shenzhen Star Instrument Co. Ltd., China). In addition, the two populations were evaluated for three other yield traits including number of panicles per plant (NP), number of grains per panicle (NGP) and grain yield per plant (GY, g).

**Table 1:** Rice populations and field experiments

Generation	Name	Segregating region		Number of lines^a^		Location and
		Marker	Physical position	NIL^ZS97^	NIL^MY46^	growing season^b^
BC_2_F_11:12_	L1	RM11807 − RM11842	34,722,600–35,694,183	41	37	HZ: May−Sep. 2014
	L2	RM265 − RM11842	35,197,724–35,694,183	21	22	HZ: May−Sep. 2014
BC_2_F_13:14_	D1	RM265 − Wn35518	35,197,724–35,518,354	25	27	HZ: May−Sep. 2016
	D2	RM265 − Wn35618	35,197,724–35,618,264	28	27	HZ: May−Sep. 2016
	D3	RM265 − RM11842	35,197,724–35,694,183	25	27	HZ: May−Sep. 2016
	D4	RM11828 − RM11842	35,315,714–35,694,183	23	25	HZ: May−Sep. 2016
	D5	Wn35518 − RM11842	35,518,508–35,694,183	25	28	HZ: May−Sep. 2016
BC_2_F_14:15_	Q1	RM265 − Wn35263	35,197,724–35,263,529	24	26	LS: Dec. 2016 − Apr. 2017
	Q2	RM265 − Wn35393	35,197,724–35,393,538	27	25	LS: Dec. 2016 − Apr. 2017
	Q3	Wn35263 − Wn35518	35,263,716–35,518,354	29	30	LS: Dec. 2016 − Apr. 2017
BC_2_F_15:16_	B1	RM265 − Wn35263	35,197,724–35,263,529	36	35	HZ: May−Sep. 2017
	B2	RM11824 − Wn35393	35,240,934–35,393,538	32	34	HZ: May−Sep. 2017

^a^NIL^ZS97^ and NIL^MY46^ are near isogenic lines with Zhenshan 97 and Milyang 46 homozygous genotypes in the segregating region, respectively

^b^HZ, Hangzhou, Zhejiang Province; LS, Lingshui, Hainan Province

### Microscopy observation

For *qGS1-35.2* that was mapped within a 57.7-kb region in this study, NIL^ZS97^ and NIL^MY46^ were taken from population B1 and used for observation of outer glume epidermal cell. Young spikelet hulls were fixed with 2.5% glutaraldehyde for 24 h and then dehydrated by a graded series of ethanol. The dehydrated sample were coated with gold-palladium using ion sputter (Model E-1010, Hitachi, Japan) and observed using scanning electron microscope (Model TM-1000, Hitachi, Japan). Cell length and width of the outer glumes were measured, and cell number in the longitudinal direction was counted. For each NIL, 20 glumes from 20 plants were used.

### DNA marker genotyping, sequence analysis and quantitative real-time PCR analysis

For population development and QTL mapping, total DNA was extracted using 2 cm-long leaf sample following the method of Zheng et al. (1995). PCR amplification was performed according to Chen et al. (1997). The products were visualized on 6% non-denaturing polyacrylamide gels using silver staining. A total of 24 polymorphic DNA markers were used, including 11 simple sequence repeat (SSR) and 13 InDel markers (Additional file 1: Table S1). The SSR markers were selected from the Gramene database (http://www.gramene.org), and the InDel markers were designed according to the differences between ZS97 and MY46 detected by whole genome re-sequencing.

Sequence analysis was performed for six annotated genes located in the target QTL region. DNA was extracted using DNeasy Plant Mini Kit (QIAGEN, German) according to the manufacturer’s instructions. The primers were designed according to the sequence of Nipponbare in RAP-DB (http://rapdb.lab.nig.ac.jp/:IRGSP-1.0) (Additional file 1: Table S2). Products amplified from the genomic DNA of ZS97 and MY46 were sequenced. Nucleotide sequence and the predicted amino acid sequence between ZS97 and MY46 were compared.

Panicles of 1 cm and 8 cm long were collected from NIL^ZS97^ and NIL^MY46^ in population B1. Total RNA was extracted using RNeasy Plus Mini Kit (QIAGEN, German). First-strand cDNA was synthesized using ReverTra AceR Kit (TOYOBO, Japan). Quantitative real-time PCR was performed on Applied Biosystems 7500 using SYBR qPCR Mix Kit (TOYOBO, Japan) according to the manufacturer’s instructions. *Actin1* was used as the endogenous control. The data were analyzed according to the 2^-ΔΔ*C*t^ method (Livak and Schmittgen, 2001). Three biological replicates and three technical replicates were used. The primers were listed in Additional file 1: Table S3.

### Data analysis

Two-way analysis of variance (ANOVA) was performed to test the phenotypic differences between the two genotypic groups in each NIL set. The analysis was performed using the SAS procedure GLM (SAS Institute 1999) as described previously (Dai et al., 2008). Given the detection of a significant difference (*P* < 0.05), the same data were used to estimate the genetic effect of the QTL, including additive effect and the proportion of phenotypic variance explained (*R*^*2*^). QTL were designated according to the rules recommended by McCouch and CGSNL (2008) with slight modification. Physical position of the first segregating marker in the QTL region was used as the unique identifier for the given QTL. For example, *qGW1-35.5* indicates that this QTL was associated with grain width (GW) and mapped in a region on chromosome 1 with the first segregating marker located at 35.5 Mb.

Cell length, width and number, as well as the expression level, were presented in mean ± s.e.m. Differences between NIL^ZS97^ and NIL^MY46^ were tested by student’s *t*-test.

## Results

### Delimitation of *qTGW1.2c* from 2.1-Mb to 1.1-Mb by increasing marker density

**Fig. 2:**
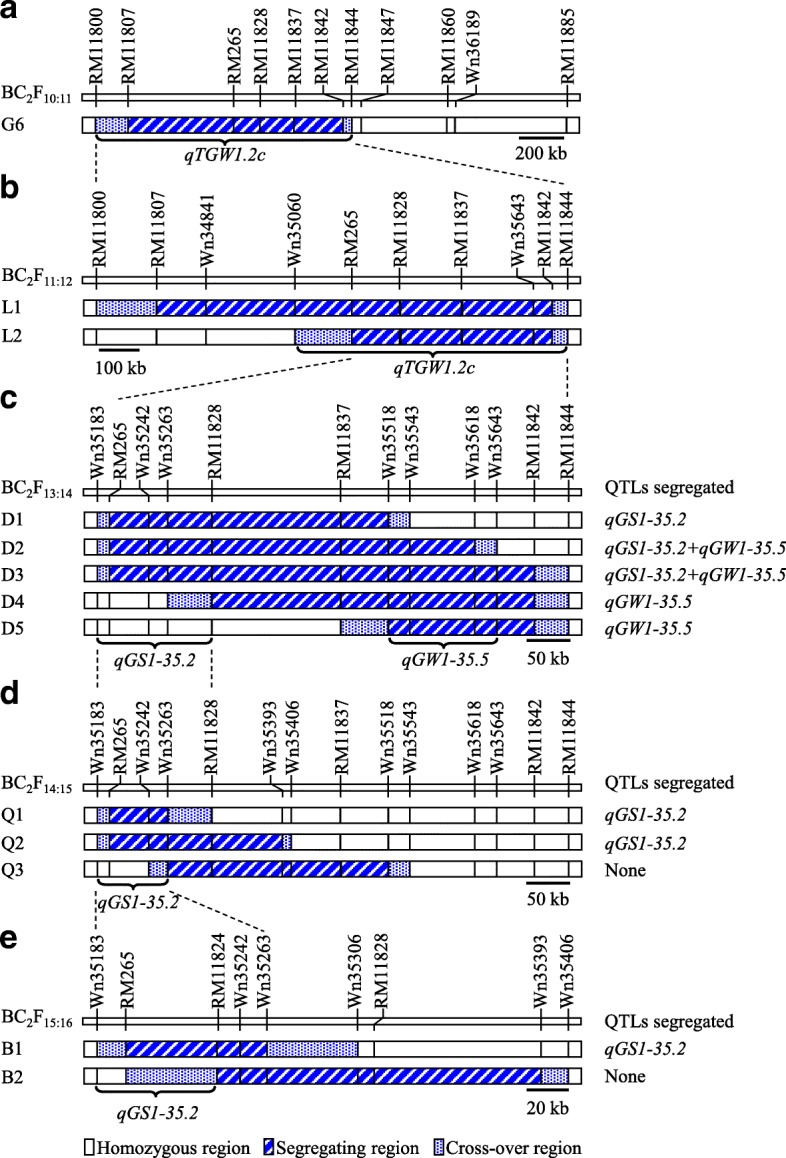
Genotypic compositions of the near isogenic lines (NILs) in target regions. **a** Composition of NIL set G6 used by Wang et al. (2015c), updated in this study with more polymorphic markers. **b** Two sets of NILs in BC_2_F_11:12_. **c** Five sets of NILs in BC_2_F_13:14_. **d** Three sets of NILs in BC_2_F_14:15_. **e** Two sets of NILs in BC_2_F_15:16_

In a previous study (Wang et al., 2015c), *qTGW1.2c* controlling grain weight in rice was located within a 2.1-Mb region between RM11800 and RM11885 on the long arm of chromosome 1. This interval included the segregating region RM11807–RM265 and two flanking cross-over regions, i.e., RM11800–RM11807 and RM265–RM11885. Based on sequence differences between ZS97 and MY46 detected by whole genome re-sequencing, seven polymorphic markers were developed. They were all located in one of the two cross-over regions RM265–RM11885. The original NIL population segregating *qTGW1.2c* was assayed using these markers. Four markers neighboring to RM11885 were homozygous, thus the downstream boundary of the QTL was moved from RM11885 to RM11844 (Fig. 2a). Therefore, *qTGW1.2c* was narrowed down to a 1.1-Mb region flanked by RM11800 and RM11844.

### Dissection of *qTGW1.2c* into two QTL

Two NIL sets were developed following the updated location of *qTGW1.2c* (Fig. 2b). Highly significant genotypic effects (*P* < 0.001) were detected for TGW and GW in both populations, with the enhancing alleles all derived from MY46 (Table 2). In L1, the additive effects were 0.20 g for TGW and 0.027 mm for GW, explaining 17.6 and 49.3% of the phenotypic variance, respectively. In L2, the additive effects were 0.22 g for TGW and 0.025 mm for GW, contributing 21.6 and 44.7% to the phenotypic variance, respectively. An opposite small effect was detected for GL, which was significant (*P* = 0.0015) in L1 only. The ZS97 allele increased GL by 0.017 mm, explaining 7.7% of the phenotypic variance. Obviously, *qTGW1.2c* affected grain weight mainly through grain width. For RLW, the ratio of GL to GW, highly significant effects (*P* < 0.0001) were detected in both populations (Table 2). The *R*^*2*^ values were 57.2 and 58.6%, higher than the values estimated for GL and GW.

**Table 2:** Validation of *qTGW1.2c* using two sets of near isogenic lines in BC_2_F_11:12_

Name	Trait^a^	Phenotype (mean ± sd)^b^		*P*	*A* ^*c*^	*R*^*2*^(%)^d^
		NIL^ZS97^	NIL^MY46^			
L1	TGW	28.67 ± 0.34	29.08 ± 0.32	< 0.0001	0.20	17.6
	GL	8.458 ± 0.040	8.423 ± 0.051	0.0015	−0.017	7.7
	GW	3.218 ± 0.021	3.272 ± 0.016	< 0.0001	0.027	49.3
	RLW	2.628 ± 0.017	2.575 ± 0.015	< 0.0001	−0.027	57.2
L2	TGW	28.86 ± 0.35	29.30 ± 0.35	0.0002	0.22	21.6
	GL	8.517 ± 0.044	8.502 ± 0.050	0.3107		
	GW	3.262 ± 0.017	3.311 ± 0.020	< 0.0001	0.025	44.7
	RLW	2.611 ± 0.012	2.568 ± 0.016	< 0.0001	−0.022	58.6

^a^*TGW* 1000-grain weight (g), *GL* grain length (mm), *GW* grain width (mm), *RLW* ratio of grain length to width

^b^NIL^ZS97^ and NIL^MY46^ are near isogenic lines with Zhenshan 97 and Milyang 46 homozygous genotypes in the segregating region, respectively

^c^Additive effect of replacing a Zhenshan 97 allele with a Milyang 46 allele

^d^Proprotion of phenotypic variance explained by the QTL effect

As described above, the effects detected in the two populations were similar, indicating that *qTGW1.2c* was located in the common segregating regions of L1 and L2. While the whole candidate region was segregated in L1, only a portion was segregated in L2. Thus, *qTGW1.2c* was located in the segregating region of L2, which was a 672.3-kb region flanked by Wn35060 and RM11844 (Fig. 2b). This result was used to develop five NIL sets with sequential segregating regions jointly covering the entire QTL region (Fig. 2c).

In all the five populations, significant effects were detected on GW with the enhancing alleles always derived from MY46 (Table 3). The additive effects were 0.009, 0.021, 0.023, 0.014 and 0.015 mm in D1, D2, D3, D4 and D5, respectively. Two alternative explanations could be given to the consistent allelic direction and varied magnitudes among the five populations. Firstly, there are two QTL for GW segregated in these populations. One was segregated in D1 but not in D4 and D5, and the other was segregated in D4 and D5 but not in D1. They were both segregated in D2 and D3, thus the additive effects were higher in the two populations than D1, D4 and D5. Secondly, one single QTL was segregated in these populations but the effect was not highly stable.

**Table 3:** Dissection of *qTGW1.2c* into two QTL using five sets of near isogenic lines in BC_2_F_13:14_

Name	Trait^a^	Phenotype (mean ± sd)^b^		*P*	*A* ^c^	*R*^*2*^ (%)^d^
		NIL^ZS97^	NIL^MY46^			
D1	TGW	28.22 ± 0.50	28.24 ± 0.44	0.8500		
	GL	8.392 ± 0.042	8.338 ± 0.046	< 0.0001	−0.027	14.7
	GW	3.063 ± 0.022	3.080 ± 0.017	0.0023	0.009	11.6
	RLW	2.740 ± 0.018	2.707 ± 0.012	< 0.0001	−0.017	39.0
D2	TGW	27.10 ± 0.39	27.73 ± 0.37	< 0.0001	0.32	30.3
	GL	8.342 ± 0.039	8.332 ± 0.042	0.3473		
	GW	3.079 ± 0.023	3.121 ± 0.023	< 0.0001	0.021	33.1
	RLW	2.710 ± 0.020	2.670 ± 0.018	< 0.0001	−0.020	41.2
D3	TGW	27.22 ± 0.38	27.75 ± 0.49	< 0.0001	0.26	15.3
	GL	8.313 ± 0.051	8.298 ± 0.042	0.2498		
	GW	3.037 ± 0.020	3.084 ± 0.022	< 0.0001	0.023	39.8
	RLW	2.738 ± 0.016	2.691 ± 0.017	< 0.0001	−0.023	49.1
D4	TGW	27.71 ± 0.39	28.11 ± 0.40	0.0013	0.20	14.3
	GL	8.286 ± 0.050	8.321 ± 0.052	0.0205	0.018	8.3
	GW	3.087 ± 0.017	3.116 ± 0.021	< 0.0001	0.014	25.7
	RLW	2.684 ± 0.018	2.671 ± 0.017	0.0192	−0.007	8.9
D5	TGW	27.25 ± 0.36	27.53 ± 0.42	0.0126	0.14	7.2
	GL	8.339 ± 0.047	8.368 ± 0.049	0.0328	0.014	5.5
	GW	3.046 ± 0.015	3.075 ± 0.016	< 0.0001	0.015	27.3
	RLW	2.738 ± 0.019	2.721 ± 0.016	0.0015	−0.008	11.7

^a^*TGW* 1000-grain weight (g), *GL* grain length (mm), *GW* grain width (mm), *RLW* ratio of grain length to width

^b^NIL^ZS97^ and NIL^MY46^ are near isogenic lines with Zhenshan 97 and Milyang 46 homozygous genotypes in the segregating region, respectively

^c^Additive effect of replacing a Zhenshan 97 allele with a Milyang 46 allele

^d^Proprotion of phenotypic variance explained by the QTL effect

For GL, significant effects were detected in three populations (Table 3). The enhancing alleles were also derived from MY46 in D4 and D5, but from ZS97 in D1. These results indicate that two QTL for GL were located in the target region. One was segregated in D1 but not in D4 and D5, and the other was segregated in D4 and D5 but not in D1. The two QTL were both segregated in D2 and D3, thus the effect became nonsignificant due to opposite directions. Taking the results on GL and GW together, it could be concluded that two QTL simultaneously affecting the two traits were segregated in these populations. One was located in a region that was segregated in D1, D2 and D3 but homozygous in D4 and D5, with the allele from MY46 decreasing GL but increasing GW (Fig. 2c; Additional file 2: Figure S1). The other was located in a region that was segregated in D2, D3, D4 and D5 but homozygous in D1, with the allele from MY46 increasing GW and GL (Additional file 3: Figure S2).

The first QTL was located within the Wn35183−RM11828 interval that corresponds to a 132.4-kb region of the Nipponbare genome (Fig. 2c). It had little effect on TGW but significantly affected GL, GW and RLW. In the D1 population that segregated this QTL only, the MY46 allele decreased GL by 0.027 mm, increased GW by 0.009 mm, and decreased RLW by 0.017, having *R*^*2*^ of 14.7, 11.6 and 39.0%, respectively (Table 3). Because this QTL primarily contributed to grain shape with the first segregating marker RM265 located at 35.2 Mb on chromosome 1, we designated it *qGS1-35.2* (Fig. 2c).

The other QTL was located in a 125.5-kb region flanked by Wn35518 and Wn35643 (Fig. 2c). It affected GL and GW with the same allelic direction and exerted significant influence on TGW. In the D4 and D5 populations that segregated this QTL only, the MY46 allele increased TGW by 0.20 and 0.14 g, GL by 0.018 and 0.014 mm, and GW by 0.014 and 0.015 mm, having *R*^*2*^ of 14.3 and 7.2%, 8.3 and 5.5%, and 25.7 and 27.3%, respectively (Table 3). Because this QTL mainly contributed to grain width with the first segregating marker Wn35518 located at 35.5 Mb on chromosome 1, we designated it *qGW1-35.5* (Fig. 2c).

### Fine-mapping of *qGS1-35.2*

One QTL, *qGS1-35.2*, was selected for further analysis. Two more runs of NIL construction – QTL mapping were performed. The first run of QTL mapping was conducted using three NIL sets, Q1, Q2 and Q3. Significant effects for grain shape traits were detected in Q1 and Q2 but not Q3. The QTL effects remained to be large on GL and RLW, and small or nonsignificant on TGW and GW (Table 4). The QTL location was delimited into an 80.4-kb region flanked by Wn35183 and Wn35263 (Fig. 2d). The second run was done using two NIL sets, B1 and B2. Significant effects for grain shape traits were detected in B1 but not B2. Finally, *qGS1-35.2* was mapped within a 57.7-kb region flanked by Wn35183 and RM11824 (Fig. 2e). The effect of this QTL was large on GL and RLW, small on GW, and nonsignificant on GT and yield traits including TGW, NP, NGP and GY (Table 4).

**Table 4:** Fine mapping of *qGS1-35.2* using five sets of near isogenic lines in BC_2_F_14:15_ and BC_2_F_15:16_

Generation	Name	Trait^a^	Phenotype (mean ± sd)^b^		*P*	*A* ^c^	*R*^*2*^(%)^d^
			NIL^ZS97^	NIL^MY46^			
BC_2_F_14:15_	Q1	TGW	27.66 ± 0.20	27.61 ± 0.22	0.3588		
		GL	8.032 ± 0.033	7.989 ± 0.034	< 0.0001	−0.022	26.1
		GW	3.235 ± 0.015	3.242 ± 0.015	0.0917		
		RLW	2.483 ± 0.014	2.464 ± 0.010	< 0.0001	−0.010	18.2
	Q2	TGW	27.57 ± 0.26	27.60 ± 0.26	0.7044		
		GL	8.023 ± 0.034	7.964 ± 0.027	< 0.0001	−0.029	33.4
		GW	3.222 ± 0.014	3.235 ± 0.018	0.0084	0.006	7.5
		RLW	2.490 ± 0.011	2.462 ± 0.014	< 0.0001	−0.014	34.3
	Q3	TGW	28.00 ± 0.34	28.13 ± 0.28	0.0986		
		GL	8.015 ± 0.036	8.017 ± 0.026	0.8229		
		GW	3.246 ± 0.017	3.249 ± 0.015	0.4148		
		RLW	2.470 ± 0.014	2.468 ± 0.014	0.5968		
BC_2_F_15:16_	B1	TGW	28.80 ± 0.27	28.76 ± 0.29	0.6390		
		GL	8.386 ± 0.030	8.325 ± 0.036	< 0.0001	−0.030	29.0
		GW	3.128 ± 0.017	3.141 ± 0.021	0.0070	0.006	5.2
		GT	2.193 ± 0.014	2.197 ± 0.009	0.1490		
		RLW	2.681 ± 0.011	2.651 ± 0.018	< 0.0001	−0.015	36.5
		NP	16.54 ± 1.79	16.44 ± 2.19	0.8313		
		NGP	55.60 ± 5.84	57.12 ± 8.90	0.3960		
		GY	24.28 ± 1.28	24.67 ± 1.88	0.3065		
	B2	TGW	27.94 ± 0.44	28.04 ± 0.30	0.2894		
		GL	8.438 ± 0.047	8.443 ± 0.041	0.6192		
		GW	3.055 ± 0.027	3.057 ± 0.019	0.6095		
		GT	2.204 ± 0.011	2.209 ± 0.011	0.0843		
		RLW	2.762 ± 0.021	2.760 ± 0.014	0.7448		
		NP	15.16 ± 0.93	15.42 ± 1.13	0.3456		
		NGP	69.04 ± 7.68	67.82 ± 6.95	0.5098		
		GY	24.12 ± 3.38	24.11 ± 3.44	0.9599		

^a^*TGW* 1000-grain weight (g), *GL* grain length (mm), *GW* grain width (mm), *GT* grain thickness (mm), *RLW* ratio of grain length to width, *NP* number of panicles per plant, *NGP* number of grains per panicle, *GY* grain yield per plant (g)

^b^NIL^ZS97^ and NIL^MY46^ are near isogenic lines with Zhenshan 97 and Milyang 46 homozygous genotypes in the segregating region, respectively

^c^Additive effect of replacing a Zhenshan 97 allele with a Milyang 46 allele

^d^Proprotion of phenotypic variance explained by the QTL effect

Length, width and number of the outer glume epidermal cells were compared between NIL^ZS97^ and NIL^MY46^ for *qGS1-35.2* (Additional file 4: Figure S3). Nonsignificant difference was detected on the cell length and width, but the cell number in the longitudinal direction was higher in NIL^ZS97^ than NIL^MY46^ (*P* = 0.0027). These results suggest that *qGS1-35.2* affects grain length by controlling cell division.

### Candidate genes of *qGS1-35.2*

According to the Rice Annotation Project Database (http://rapdb.dna.affrc.go.jp/), there are six annotated genes in the 57.7-kb region for *qGS1-35.2*. Three of them encode proteins containing known functional domains. *Os01g823900* encodes the U-box E3 ubiquitin ligase OsPUB3 that regulates the response to abiotic stress (Byun et al., 2017). *Os01g0824600* produces two different transcripts, encoding a serine/threonine protein kinase domain containing protein or CBL-interacting protein kinase 11 that are involved in various biological processes (Sanyal et al., 2015). *Os01g0824700* encodes a member of the cyclin-like F-box domain containing proteins that are major components of E3 ubiquitin-protein ligase and participate in a large variety of biological processes including seed development (Somers & Fujiwara, 2009, Chen et al., 2013; Gupta, Garg & Bhatia, 2015). The remaining three annotated genes are *Os01g0823951*, *Os01g0824000* and *Os01g0824500* that encode hypothetical proteins.

Sequence comparisons of the six annotated genes were conducted between full-length genomic fragments of ZS97 and MY46 (Additional file 1: Table S4). Among the three genes encoding proteins of known functional domains, no difference was identified in *Os01g0824600* but single nucleotide polymorphisms (SNPs) were detected in the other two genes. For *Os01g0824700*, the G116A substitution resulted in a premature stop codon in MY46. For *Os01g823900*, four SNPs were found, of which two were synonymous and the other two were non-synonymous. SNPs resulting in non-synonymous mutation were also found for all the three genes encoding hypothetical proteins.

Transcript levels of the six annotated genes in panicle were compared between NIL^ZS97^ and NIL^MY46^ for *qGS1-35.2* (Additional file 5: Figure S4). For the two transcripts produced by *Os01g0824600*, significant difference was only detected on *Os01t0824600-2* encoding CBL-interacting protein kinase 11. In panicle of 1 cm and 8 cm long, the expression levels were 1.8 and 1.6 times higher in NIL^MY46^ than NIL^ZS97^, respectively. Two more genes were found to have significant expression differences between the two NILs. They were *Os01g0823951* and *Os01g082400* encoding hypothetical proteins. As compared with NIL^ZS97^, the expression levels of NIL^MY46^ in panicles of 1 cm and 8 cm were 6.2 and 7.6 times higher on *Os01g0823951*, and 1.6 and 0.8 times higher on *Os01g082400*, respectively. Nonsignificant difference was detected on other genes.

## Discussion

In recent years, increasing attention has been paid to the cloning of minor QTL in rice, but none has been reported for traits determining grain size. In the present study, two minor QTL associated with grain size in rice were dissected and fine-mapped. They were located in the 460-kb interval Wn35183−Wn35643 on the long arm of chromosome 1. One of them, *qGS1-35.2*, was delimited into a 57.7-kb region flanked by Wn35183 and RM11824, affecting grain length and width with opposite allelic directions and showing little influence on grain weight. The other one, *qGW1-35.5*, was mapped within a 125.5-kb region flanked by Wn35518 and Wn35643, primarily controlling grain width and consequently affecting grain weight. Our work lays a foundation for cloning the genes underlying these two minor QTL for grain size.

Clustering of genes for the same trait is frequently observed in plant genome. This has been evident for genes having large effects on grain size in rice. For example, a 3.2-Mb region on the short arm of chromosome 3 covers five genes, including *PGL1*, *BG1*, *OsLG3*, *OsLG3b*/*qLGY3*, and *TUD1* (Heang & Sassa, 2012a; Hu et al., 2013; Li & Li, 2016; Liu et al., 2018; Yu et al., 2018); a 4.3-Mb region on the short arm of chromosome 5 covers seven genes, including *APG*, *OsPPKL2*, *SRS3*, *GS5*, *GW5/qSW5*, *GSK2* and *OsCYP51G3* (Heang & Sassa, 2012b; Zhang et al., 2012; Huang et al., 2013; Xia et al., 2015; Li & Li, 2016). Clustering of QTL having small effect on grain size in rice has also been observed. In our previous studies, five minor QTL for grain size were dissected in a region on the long arm of chromosome 1 (Wang et al., 2015c; Zhang et al., 2016). One of them, *qTGW1.2c*, was separated into two QTL in the present study. Altogether, six minor QTL for grain size have been separated in a 7.1-Mb region using one *indica* rice cross, spanning from the upstream boundary marker Wn28447 for *qTGW1.1a* (Zhang et al., 2016) to the downstream boundary marker Wn35643 for *qGW1-35.5* reported here. These results suggest that grain size in rice is controlled by a large number of QTL, including a few loci with large effects and numerous loci with small effects, which is similar to the genetic architecture of heading date in rice (Hori et al., 2015).

Grain size and shape are both determined by grain length and width. While grain size is the major determinant of grain weight, grain shape is mainly related to consuming preference (Calingacion et al., 2014) and may not be associated with grain weight. For genes having similar effects on grain length and width with opposite directions, such as *GL7*/*GW7* (Song et al., 2015), the influence is usually exerted on grain shape rather than grain weight. One of the two QTL we identified, *qGS1-35.2*, affected grain shape without influencing grain weight and other yield traits. The MY46 allele decreased grain length but increased grain width, resulting in little effect on grain weight and enhanced effect on the ratio of grain length to width (Table 3). This type of QTL could be used to modify grain shape without yield penalty. For genes simultaneously controlling grain length and width with the same allelic direction, and those contributing to either grain length or width, such as most of the cloned genes conditioning these traits (Li and Li, 2016; Yu et al., 2017), the same direction of QTL effect is always simultaneously detected on grain size and grain weight. Another QTL we identified, *qGW1-35.5*, falls into this category (Table 3). The MY46 allele significantly increased both the grain length and width, and in the meantime enhanced grain weight. This type of QTL could be used for yield improvement. More and more choices for breeding utilization can be anticipated with the identification of new genes for grain size traits.

Three annotated genes encoding proteins with known functional domains were located in the *qGS1-35.2* region. They were involved in two important pathways that regulate grain size in plants (Zuo & Li, 2014; Li & Li, 2016). *Os01g0824700* and *Os01g0823900* encode two proteins that are important components of ubiquitin ligases (Xu et al., 2009; Byun et al., 2017), and *Os01g0824600* participates in plant hormone signaling pathway (Xiang, Huang & Xiong, 2007). For *Os01g0824700*, a premature stop mutation was found in MY46, which usually fully disrupt gene function. In addition, nonsignificant difference was detected between NIL^ZS97^ and NIL^MY46^ on the expression of *Os01g0824700*. It is unlikely that this is the gene for *qGS1-35.2*. For *Os01g823900*, two amino acid substitutions were identified, which may cause minor phenotypic change as suggested in previous studies (Matsubara et al., 2012; Wu et al., 2013; Shibaya et al., 2016). For *Os01g0824600*, no difference was identified in its coding region, but expression difference was found on one of the two transcripts of this gene (Additional file 5: Figure S4). Both *Os01g823900* and *Os01g0824600* are more likely to be the gene underlying *qGS1-35.2*.

Three other annotated genes, *Os01g0823951*, *Os01g0824000* and *Os01g0824500* encoding hypothetical proteins, were also located in the *qGS1-35.2* region. All of them showed amino acid substitutions. Expression differences were also detected on two of the genes, *Os01g0823951* and *Os01g0824000*. None of them could be ruled out from the candidate genes for *qGS1-35.2*. Therefore, more work is needed to clarify which gene is the one for QTL *qGS1-35.2*.

## Conclusion

Two closely linked minor QTL for grain size in rice were separated on the long arm of chromosome 1. The *qGS1-35.2* was delimited into a 57.7-kb region in which six annotated genes were found. This QTL regulates grain length and width with opposite allelic directions, affecting grain shape but having little influence on grain weight and other yield traits, providing a potential gene resource for fine-tuning grain shape to modify grain appearance quality without yield penalty. The *qGW1-35.5* regulates grain width and length with the same allelic direction, simultaneously affecting grain shape, size and weight, offering a new gene resource for enhancing grain yield.

## Additional files

Additional file 1: **Table S1.** Primers used for population development and QTL mapping. **Table S2.** Primers used for sequence analysis. **Table S3.** Primers used for quantitative real-time PCR. **Table S4.** Nucleotide and amino acid differences between Zhenshan 97 and Milyang 46. (DOCX 23 kb)

Additional file 2: **Figure S1.** Grains of NIL^ZS97^ and NIL^MY46^ for *qGS1-35.2.* Scale bar, 20 mm. (PDF 1936 kb)

Additional file 3: **Figure S2.** Grains of NIL^ZS97^ and NIL^MY46^ for *qGW1-35.5.* Scale bar, 20 mm. (PDF 1946 kb)

Additional file 4: **Figure S3.** Characterization of the cells in outer glumes of NIL^ZS97^ and NIL^MY46^ for *qGS1-35.2*. **a** Scanning electron microscopic images of the cells. Scale bar, 200 μm. **b** Cell length, width and number. The cell numbers were measured in the longitudinal direction. Data are presented in mean ± s.e.m. (*n* = 20). A Student’s *t*-test was used to generate the *P* values. (PDF 1633 kb)

Additional file 5: **Figure S4.** Transcript levels of annotated genes in the *qGS1-35.2* region. The experiment was performed using panicles of 1 cm (P1) and 8 cm (P8) collected from NIL^ZS97^ and NIL^MY46^ for *qGS1-35.2*. The expression levels were normalized to *Actin1* and related to P1 of NIL^ZS97^. Data are presented in mean ± s.e.m. (*n* = 3). A Student’s *t*-test was used to generate the *P* values. (PDF 2579 kb)

## Abbreviations

ANOVA: Analysis of Variance
GL: Grain Length
GS: Grain Shape
GT: Grain Thickness
GW: Grain Width
GY: Grain Yield per Plant
MY46: Milyang 46
NGP: Number of Grains per Panicle
NIL: Near Isogenic Line
NP: Panicle per Plant
QTL: Quantitative Trait Locus
*R*^*2*^: Proportion of Phenotypic Variance Explained
RAP-DB: Rice Annotation Project Database
RLW: Ratio of Grain Length to Width
SNP: Single Nucleotide Polymorphism
SSR: Simple Sequence Repeat
TGW: 1000-Grain Weight
ZS97: Zhenshan 97

## References

[CR1] Byun MY, Cui LH, Oh TK, Jung Y-J, Lee A, Park KY, Kang BG, Kim WT (2017). Homologous U-box E3 ubiquitin ligases OsPUB2 and OsPUB3 are involved in the positive regulation of low temperature stress response in rice (*Oryza sativa* L.). Front Plant Sci.

[CR2] Calingacion M, Laborte A, Nelson A, Resurreccion A, Concepcin JC, Daygon VD, Mumm R, Reinke R, Dipti S, Bassinello PZ, Manful J, Sophany S, Lara KC, Bao J, Xie L, Loaiza K, EI-hissewy A, Gayin J, Sharma N, Rajeswari S, Manonmani S, Rani NS, Kota S, Indrasari SD, Habibi F, Hosseini M, Tavasoli F, Suzuki K, Umemoto T, Boualaphanh C, Lee HH, Hung YP, Ramli A, Aung PP, Ahmad R, Wattoo JI, Bandonill E, Romero M, Brites CM, Hafeel R, Lur H-S, Cheaupun K, Jongdee S, Blanco P, Bryant R, Lang NT, Hall RD, Fitzgerald M (2014). Diversity of global rice markets and the science required for consumer-targeted rice breeding. PLoS One.

[CR3] Chen J-Y, Guo L, Ma H, Chen Y-Y, Zhang H-W, Ying J-Z, Zhuang J-Y (2014). Fine mapping of *qHd1*, a minor heading date QTL with pleiotropism for yield traits in rice (*Oryza sativa* L.). Theor Appl Genet.

[CR4] Chen L, Zhong Z, Wu W, Liu L, Lu G, Jin M, Tan J, Sheng P, Wang D, Wang J, Cheng Z, Wang J, Zhang X, Guo X, Wu F, Lin Q, Zhu S, Jiang L, Zhai H, Wu C, Wan J (2015). Fine mapping of *DTH3b*, a minor heading date QTL potentially functioning upstream of *Hd3a* and *RFT1* under long-day conditions in rice. Mol Breeding.

[CR5] Chen X, Temnykh S, Xu Y, Cho YG, McCouch SR (1997). Development of a microsatellite framework map providing genome-wide coverage in rice (*Oryza sativa* L.). Theor Appl Genet.

[CR6] Chen Y, Xu Y, Luo W, Li W, Chen N, Zhang D, Chong K (2013). The F-box protein OsFBK12 targets OsSAMS1 for degradation and affects pleiotropic phenotypes, including leaf senescence, in rice. Plant Physiol.

[CR7] Dai W-M, Zhang K-Q, Wu J-R, Wang L, Duan B-W, Zheng K-L, Cai R, Zhuang J-Y (2008). Validating a segment on the short arm of chromosome 6 responsible for genetic variation in the hull silicon content and yield traits of rice. Euphytica.

[CR8] Gupta S, Garg V, Bhatia S (2015). A new set of ESTs from chickpea (*Cicer arietinum* L.) embryo reveals two novel F-box genes, *CarF-box_PP2* and *CarF-box_LysM*, with potential roles in seed development. PLoS One.

[CR9] Heang D, Sassa H (2012). An atypical bHLH protein encoded by *POSITIVE REGULATOR OF GRAIN LENGTH 2* is involved in controlling grain length and weight of rice through interaction with a typical bHLH protein APG. Breeding Sci.

[CR10] Heang D, Sassa H (2012). Antagonistic actions of HLH/bHLH proteins are involved in grain length and weight in rice. PLoS One.

[CR11] Hori K, Nonoue Y, Ono N, Shibaya T, Ebana K, Matsubara K, Ogiso-Tanaka E, Tanabata T, Sugimoto K, Taguchi-Shiobara F, Yonemaru J, Mizobuchi R, Uga Y, Fukuda A, Ueda T, Yamamoto S, Yamanouchi U, Takai T, Ikka T, Kondo K, Hoshino T, Yamamoto E, Adachi S, Nagasaki H, Shomura A, Shimizu T, Kono I, Ito S, Mizubayashi T, Kitazawa N, Nagata K, Ando T, Fukuoka S, Yamamoto T, Yano M (2015). Genetic architecture of variation in heading date among Asian rice accessions. BMC Plant Biol.

[CR12] Hu X, Qian Q, Xu T, Zhang Y, Dong G, Gao T, Xie Q, Xue Y (2013). The U-box E3 ubiquitin ligase TUD1 functions with a heterotrimeric G *α* subunit to regulate brassinosteroid-mediated growth in rice. PLoS Genet.

[CR13] Huang R, Jiang L, Zheng J, Wang T, Wang H, Huang Y, Hong Z (2013). Genetic bases of rice grain shape: so many genes, so little known. Trends Plant Sci.

[CR14] Li N, Li Y (2016). Signaling pathways of seed size control in plants. Curr Opin Plant Biol.

[CR15] Liu Q, Han R, Wu K, Zhang J, Ye Y, Wang S, Chen J, Pan Y, Li Q, Xu X, Zhou J, Tao D, Wu Y, Fu X (2018). G-protein βγ subunits determine grain size through interaction with MADS-domain transcription factors in rice. Nat Commun.

[CR16] Livak KJ, Schmittgen TD (2001). Analysis of relative gene expression data using real-time quantitative PCR and the 2^-ΔΔ*C*t^ method. Methods.

[CR17] Mackay TFC, Stone EA, Ayroles JF (2009). The genetics of quantitative traits: challenges and prospects. Nat Rev Genet.

[CR18] Matsubara K, Ogiso-Tanaka E, Hori K, Ebana K, Ando T, Yano M (2012). Natural variation in *Hd17*, a homolog of *Arabidopsis ELF3* that is involved in rice photoperiodic flowering. Plant Cell Physiol.

[CR19] Mc Couch SR, CGSNL (Committee on Gene Symbolization, Nomenclature and Linkage, Rice Genetics Cooperative) (2008). Gene nomenclature system for rice. Rice.

[CR20] Sanyal S, Pandey A, Pandey GK (2015). The CBL–CIPK signaling module in plants: a mechanistic perspective. Physiol Plantarum.

[CR21] SAS Institute Inc (1999) SAS/STAT user’s guide. Cary. SAS Institute

[CR22] Shibaya T, Hori K, Ogiso-Tanaka E, Yamanouchi U, Shu K, Kitazawa N, Shomura A, Ando T, Ebana K, Wu J, Yamazaki T, Yano M (2016). *Hd18*, encoding histone acetylase related to Arabidopsis FLOWERING LOCUS D, is involved in the control of flowering time in rice. Plant Cell Physiol.

[CR23] Somers DE, Fujiwara S (2009). Thinking outside the F-box: novel ligands for novel receptors. Trends Plant Sci.

[CR24] Song XJ, Kuroha T, Ayano M, Furuta T, Nagai K, Komeda N, Segami S, Miura K, Ogawa D, Kamura T, Suzuki T, Higashiyama T, Yamasaki M, Mori H, Inukai Y, Wu J, Kitano H, Sakakibara H, Jacobsen SE, Ashikari M (2015). Rare allele of a previously unidentified histone H4 acetyltransferase enhances grain weight, yield, and plant biomass in rice. P Natl Acad Sci USA.

[CR25] Wang L-L, Chen Y-Y, Guo L, Zhang H-W, Fan Y-Y, Zhuang J-Y (2015). Dissection of *qTGW1.2* to three QTLs for grain weight and grain size in rice (*Oryza sativa* L.). Euphytica.

[CR26] Wang S, Li S, Liu Q, Wu K, Zhang J, Wang S, Wang Y, Chen X, Zhang Y, Gao C, Wang F, Huang H, Fu X (2015). The *OsSPL16*-*GW7* regulatory module determines grain shape and simultaneously improves rice yield and grain quality. Nat Genet.

[CR27] Wang Y, Xiong G, Hu J, Jiang L, Yu H, Xu J, Fang Y, Zeng L, Xu E, Xu J, Ye W, Meng X, Liu R, Chen H, Jing Y, Wang Y, Zhu X, Li J, Qian Q (2015). Copy number variation at the *GL7* locus contributes to grain size diversity in rice. Nat Genet.

[CR28] Wu W, Liu X, Wang M, Meyer RS, Luo X, Ndjiondjop M-N, Tan L, Zhang J, Wu J, Cai H, Sun C, Wang X, Wing RA, Zhu Z (2017). A single-nucleotide polymorphism causes smaller grain size and loss of seed shattering during African rice domestication. Nat Plants.

[CR29] Wu W, Zheng X-M, Lu G, Zhong Z, Gao H, Chen L, Wu C, Wang H, Wang Q, Zhou K, Wang J-L, Wu F, Zhang X, Guo X, Cheng Z, Lei C, Lin Q, Jiang L, Wang H, Ge S, Wan J (2013). Association of functional nucleotide polymorphisms at *DTH2* with the northward expansion of rice cultivation in Asia. P Natl Acad Sci USA.

[CR30] Xia K, Ou X, Tang H, Wang R, Wu P, Jia Y, Wei X, Xu X, Kang S-H, Kim S-K, Zhang M (2015). Rice microRNA Osa-miR1848 targets the obtusifoliol 14α-demethylase gene *OsCYP51G3* and mediates the biosynthesis of phytosterols and brassinosteroids during development and in response to stress. New Phytol.

[CR31] Xiang Y, Huang Y, Xiong L (2007). Characterization of stress-responsive *CIPK* genes in rice for stress tolerance improvement. Plant Physiol.

[CR32] Xu G, Ma H, Nei M, Kong H (2009). Evolution of F-box genes in plants: different modes of sequence divergence and their relationships with functional diversification. P Natl Acad Sci USA.

[CR33] Yan S, Zou G, Li S, Wang H, Liu H, Zhai G, Guo P, Song H, Yan C, Tao Y (2011). Seed size is determined by the combinations of the genes controlling different seed characteristics in rice. Theor Appl Genet.

[CR34] Yano M, Katayose Y, Ashikari M, Yamanouchi U, Monna L, Fuse T, Baba T, Yamamoto K, Umehara Y, Nagamura Y, Sasaki T (2000). *Hd1*, a major photoperiod sensitivity quantitative trait locus in rice, is closely related to the Arabidopsis flowering time gene *CONSTANS*. Plant Cell.

[CR35] Yu J, Miao J, Zhang Z, Xiong H, Zhu X, Sun X, Pan Y, Liang Y, Zhang Q, Rashid MAR, Li J, Zhang H, Li Z (2018) Alternative splicing of *OsLG3b* controls grain length and yield in *japonica* rice. Plant Biotechnol J. 10.1111/pbi.12903 10.1111/pbi.12903 PMC6097128 29479793

[CR36] Yu J, Xiong H, Zhu X, Zhang H, Li H, Miao J, Wang W, Tang Z, Zhang Z, Yao G, Zhang Q, Pan Y, Wang X, Rashid MAR, Li J, Gao Y, Li Z, Yang W, Fu X, Li Z (2017). *OsLG3* contributing to rice grain length and yield was mined by ho-LAMap. BMC Biol.

[CR37] Zhang H-W, Fan Y-Y, Zhu Y-J, Chen J-Y, Yu S-B, Zhuang J-Y (2016). Dissection of the *qTGW1.1* region into two tightly-linked minor QTLs having stable effects for grain weight in rice. BMC Genet.

[CR38] Zhang X, Wang J, Huang J, Lan H, Wang C, Yin C, Wu Y, Tang H, Qian Q, Li J, Zhang H (2012). Rare allele of *OsPPKL1* associated with grain length causes extra-large grain and a significant yield increase in rice. P Natl Acad Sci USA.

[CR39] Zheng KL, Huang N, Bennett J, Khush GS (1995) PCR-based marker-assisted selection in rice breeding: IRRI Discussion Paper Series No. 12. Los Banos: International Rice Research Institute

[CR40] Zhong Z, Wu W, Wang H, Chen L, Liu L, Wang C, Zhao Z, Lu G, Gao H, Wei X, Yu C, Chen M, Shen Y, Zhang X, Cheng Z, Wang J, Jiang L, Wan J (2014). Fine mapping of a minor-effect QTL, *DTH12*, controlling heading date in rice by up-regulation of florigen genes under long-day conditions. Mol Breeding.

[CR41] Zuo J, Li J (2014). Molecular genetic dissection of quantitative trait loci regulating rice grain size. Annu Rev Genet.

