# Supplementary material for: Dissection and fine-mapping of two QTL for grain size linked in a 460-kb region on chromosome 1 of rice
Source: Rice (N Y). 2018 Aug 2;11:44. doi: 10.1186/s12284-018-0236-z (PMC6081826; doi:10.1186/s12284-018-0236-z)
Supplement: Supplementary file 1 — Table S1. Primers used for population development and QTL mapping. Table S2. Primers used for sequence analysis. Table S3. Primers used for quantitative real-time PCR. Table S4. Nucleotide and amino acid differences between Zhenshan 97 and Milyang 46. (DOCX 23 kb) [file 12284_2018_236_MOESM1_ESM.docx]

**Additional file 1: Table S1** Primers used for population development and QTL mapping.

| Name | Type | Forward primer (5'-3') | Physical position | | Reverse primer (5'-3') | Physical position | |
| --- | --- | --- | --- | --- | --- | --- | --- |
|  |  |  | Start | Stop |  | Start | Stop |
| RM11800 | SSR | GTCTGTTGTCTTTGTTGCCAAGG | 34,574,766 | 34,574,788 | CTTCCTGTGCTTACTCTTCTTCTTGC | 34,574,965 | 34,574,990 |
| RM11807 | SSR | CTACGTTTGGATCGGAGGAGAGG | 34,722,486 | 34,722,508 | CCCTTCTATTTCGCTTCCATTTCC | 34,722,577 | 34,722,600 |
| Wn34841 | InDel | GAACTTATTATTTTAGACGGAACGG | 34,841,259 | 34,841,283 | AGAAGTCAAAAGGGCCACGA | 34,841,363 | 34,841,382 |
| Wn35060 | InDel | ATATTGGAAGACGAGACTAGAAGCC | 35,060,894 | 35,060,918 | TCATTAGTTCTAAATTGTATATCGTCA | 35,061,055 | 35,061,081 |
| Wn35183 | InDel | ATGGTGTCAACCCCAA | 35,182,928 | 35,182,943 | AGTCAACGGGTTATTCATGC | 35,183,105 | 35,183,124 |
| RM265 | SSR | CGAGTTCGTCCAAGTGAGC | 35,197,706 | 35,197,724 | CATCCACCATTCCACCAATC | 35,197,616 | 35,197,635 |
| RM11824 | SSR | GTACTGCAAATCTAGATGGACTGG | 35,240,818 | 35,240,841 | TAGCTATTCATCTGCCACAAGC | 35,240,913 | 35,240,934 |
| Wn35242 | InDel | TAACCAAAGTCAAAACGTC | 35,242,134 | 35,242,152 | CCTTATATTACGGGACTGA | 35,242,293 | 35,242,311 |
| Wn35263 | InDel | CCAAACCATACTAACGC | 35,263,529 | 35,263,545 | GCAGTGACTCTTTAGC | 35,263,701 | 35,263,716 |
| Wn35306 | InDel | ACAGTGCAGCAAGAGATACCAT | 35,306,570 | 35,306,591 | GTGCAGATCCAGAGAACAGTGAC | 35,306,707 | 35,306,729 |
| RM11828 | SSR | TTCTGCAGATGCACAATGAAGC | 35,315,524 | 35,315,545 | GACAATGTTGAGGCTCCTAAAGAGG | 35,315,690 | 35,315,714 |
| Wn35393 | InDel | AACAACCTCCCGATCCTCA | 35,393,538 | 35,393,556 | TTTTCTTTATGAAGGCGCTCT | 35,393,714 | 35,393,734 |
| Wn35406 | InDel | ATAATTCCTTCCCCACATATTCG | 35,406,297 | 35,406,319 | TAACGGATTTCCTAAATTTATGGC | 35,406,422 | 35,406,445 |
| RM11837 | SSR | CCATGACCTACACAAACGAAACG | 35,472,004 | 35,472,026 | ATCAATCGCCACTCAATCTCTGC | 35,472,276 | 35,472,298 |
| Wn35518 | InDel | CCCTCGATCTAAACACC | 35,518,354 | 35,518,370 | TAAATTTATCTTAGGTCGAAA | 35,518,488 | 35,518,508 |
| Wn35543 | InDel | CGAGGTTCATGTCACATCT | 35,542,976 | 35,542,994 | CCTCAGTTGTAGACGCAAT | 35,543,142 | 35,543,160 |
| Wn35618 | InDel | TTGTCCCTGAGTGTTTCGGA | 35,618,264 | 35,618,283 | GACTTGTTACACGTTTCTAGCTT | 35,618,437 | 35,618,459 |
| Wn35643 | InDel | ACACACAAAGTTGAGAGAGCG | 35,643,998 | 35,644,018 | AGGAACAAAACAAGTAAAGTGGAT | 35,644,096 | 35,644,119 |
| RM11842 | SSR | CCAAACAATTGGTAGTGTCG | 35,694,183 | 35,694,202 | TTTCTAGTCTCCACAGTGAACG | 35,694,299 | 35,694,320 |
| RM11844 | SSR | TGTGCTATTGCGCGAGATTATCC | 35,733,354 | 35,733,376 | CATCACCGCTCCAACTCATGC | 35,733,577 | 35,733,597 |
| RM11847 | SSR | TCTTCATTGCATTGCTGCTACG | 35,770,021 | 35,770,042 | AACCAACCGTACTGTTTCTTCTCC | 35,770,276 | 35,770,299 |
| RM11860 | SSR | GTGATAGCAGATGAGCAGCATGG | 36,154,588 | 36,154,610 | AGTAACAGCTGCCACAACTTTGC | 36,154,697 | 36,154,719 |
| Wn36189 | InDel | CATATATAATACCCGATGTAACACGCTA | 36,189,978 | 36,190,005 | AGTGAGGAGCCTTTAATTCACAACAAC | 36,190,107 | 36,190,133 |
| RM11885 | SSR | ACACCCTTCATTTGCGTGTATGG | 36,687,673 | 36,687,695 | GGCACCTTCGACTTTGTAAATGC | 36,687,830 | 36,687,852 |

**Table S2** Primers used for sequence analysis.

| Name | Forward primer (5'-3') | Physical position | | Reverse primer (5'-3') | Physical position | |
| --- | --- | --- | --- | --- | --- | --- |
|  |  | Start | Stop |  | Start | Stop |
| S0823900 | CCAATCGTCGCAATCACTCC | 35,185,257 | 35,185,276 | CCCTTTCCAGCCAATGCATC | 35,188,123 | 35,188,142 |
| S0823951 | TGCGTACACAATTCTCATAAACC | 35,191,667 | 35,191,689 | GGTCGTCCGAATTACTCCCT | 35,193,449 | 35,193,468 |
| S0824000 | CCGTCGAAAATTTACATTCACT | 35,191,876 | 35,191,897 | AGCTCATCTTGCACTCTCAA | 35,194,889 | 35,194,908 |
| S0824500 | ACTAGTCACGTAGTAGTTCCC | 35,224,444 | 35,224,464 | CCATGTATACCACGTCAGCAA | 35,226,918 | 35,226,938 |
| S0824600 | GCCAGCGATATTTACCTGTCC | 35,228,786 | 35,228,806 | TCCTTGCTCTATGTCCGAAC | 35,234,609 | 35,234,628 |
| S0824700 | CCTTCTCCCTGTGTGCAAG | 35,237,794 | 35,237,812 | AGCTCTCGCAAATCGTAACACC | 35,240674 | 35,240,695 |

**Table S3** Primers used for quantitative real-time PCR.

| Name | Forward primer (5'-3') | | Reverse primer (5'-3') |  |
| --- | --- | --- | --- | --- |
| Q0823900 | GCTGAACAGGACGCACTTGG | TGCCTCGGAGAAATGGACG | | |
| Q0823951 | CGACTCTTGGGCACAATGGC | GCACAAAACGACGATGAACGAG | | |
| Q0824000 | CCTGGCAGAGCAGCAAGCA | GTGGGTCGCGTAGCGGAAT | | |
| Q0824500 | GCGTGATGATGGCCTACTGG | CCTTCTCGCTTCCCTTTGC | | |
| Q0824600-1 | TGACGACGACGGATAACA | TGAGCAAACACCACCACA | | |
| Q0824600-2 | GTGTCGCTTCTGGTTGCT | TATGGGCTGGAGGGAGTA | | |
| Q0824700 | TGTACGCTGTCAAACCAA | CCTCTTCTGTGATAGTCCC | | |

**Table S4** Nucleotide and amino acid differences between Zhenshan 97 and Milyang 46

| Locus name | Polymorphism using Zhenshan 97 as reference^a^ | | | | |  |  |  |  |  | NCBI accessions | |
| --- | --- | --- | --- | --- | --- | --- | --- | --- | --- | --- | --- | --- |
|  |  |  |  |  |  |  |  |  |  |  | Zhenshan 97 | Milyang 46 |
| Os01g0823900 | NP | C97T | T438C | A1546T | C1995T |  |  |  |  |  | MH018591 | MH018592 |
|  | AP | P33L | L146L | T516S | G665G |  |  |  |  |  |  |  |
| Os01g0823951 | NP | G62A | G87C | G118A |  |  |  |  |  |  | MH018595 | MH018596 |
|  | AP | R21Q | G39G | R40H |  |  |  |  |  |  |  |  |
| Os01g0824000 | NP | A80G | G121A | G170T | T295C | G297A | T360A | C436G | C474T | G514A | MH018593 | MH018594 |
|  | AP | H27R | E41R | R57L | W99R | W99R | P120P | P146A | S158S | A172T |  |  |
| Os01g0824500 | NP | C363A | C446T | A747G | G1040A | A1074G |  |  |  |  | MH018589 | MH018590 |
|  | AP | intron | intron | intron | R81Q | P92P |  |  |  |  |  |  |
| Os01g0824600 | NP | no difference | |  |  |  |  |  |  |  | MH018597, MH018599 | MH018598, MH018600 |
|  | AP | no difference | |  |  |  |  |  |  |  |  |  |
| Os01g0824700 | NP | T71C | G116A | A178G | C591G | C793G | C1033T |  |  |  | MH018587 | MH018588 |
|  | AP | P23P | W39stop | - | - | - | - |  |  |  |  |  |

^a^NP, Nucleotide polymorphism; AP, Amino acid polymorphism
