# Supplementary figures and images for: Dissection and fine-mapping of two QTL for grain size linked in a 460-kb region on chromosome 1 of rice
Source: Rice (N Y). 2018 Aug 2;11:44. doi: 10.1186/s12284-018-0236-z (PMC6081826; doi:10.1186/s12284-018-0236-z)

**NIL**<sup>ZS97</sup>

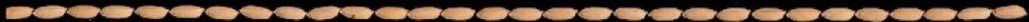

**NIL**<sup>MY46</sup>

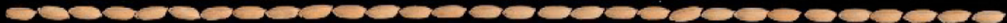

**NIL**<sup>ZS97</sup>

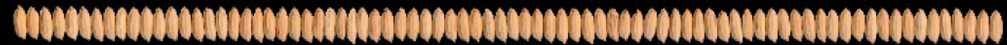

**NIL**<sup>MY46</sup>

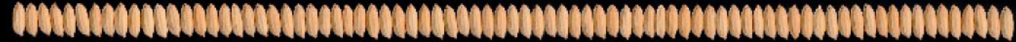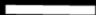

Supplement: Supplementary file 2 — Figure S1. Grains of NILZS97 and NILMY46 for qGS1-35.2. Scale bar, 20 mm. (PDF 1936 kb) [file 12284_2018_236_MOESM2_ESM.pdf]

NIL<sup>ZS97</sup>

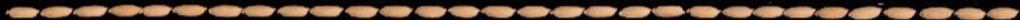

NIL<sup>MY46</sup>

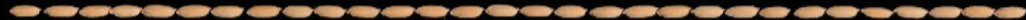

NIL<sup>ZS97</sup>

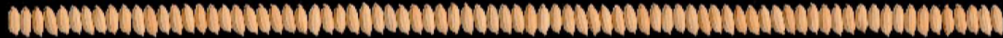

NIL<sup>MY46</sup>

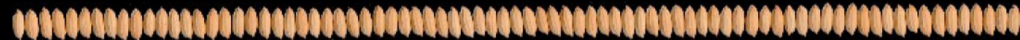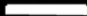

Supplement: Supplementary file 3 — Figure S2. Grains of NILZS97 and NILMY46 for qGW1-35.5. Scale bar, 20 mm. (PDF 1946 kb) [file 12284_2018_236_MOESM3_ESM.pdf]

**a**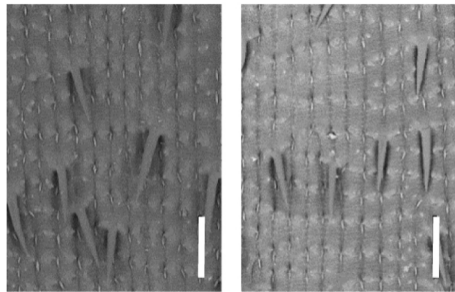 $NIL^{ZS97}$  $NIL^{MY46}$ **b**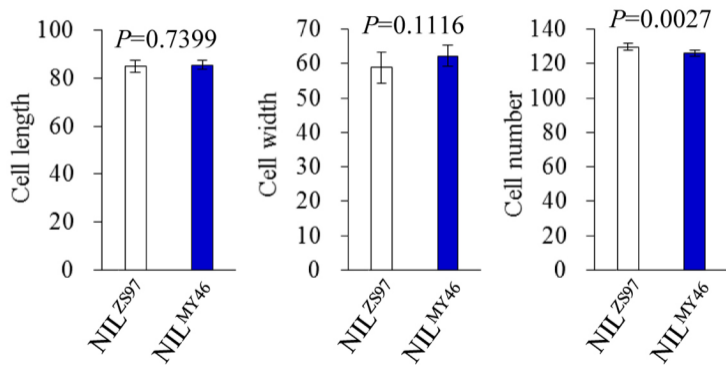

Supplement: Supplementary file 4 — Figure S3. Characterization of the cells in outer glumes of NILZS97 and NILMY46 for qGS1-35.2. a Scanning electron microscopic images of the cells. Scale bar, 200 μm. b Cell length, width and number. The cell numbers were measured in the longitudinal direction. Data are presented in mean ± s.e.m. (n = 20). A Student’s t-test was used to generate the P values. (PDF 1633 kb) [file 12284_2018_236_MOESM4_ESM.pdf]

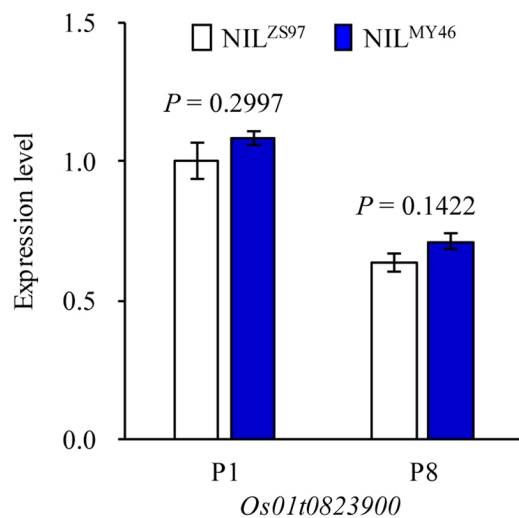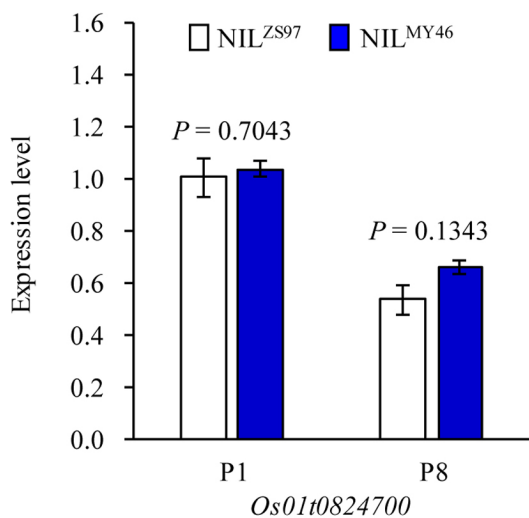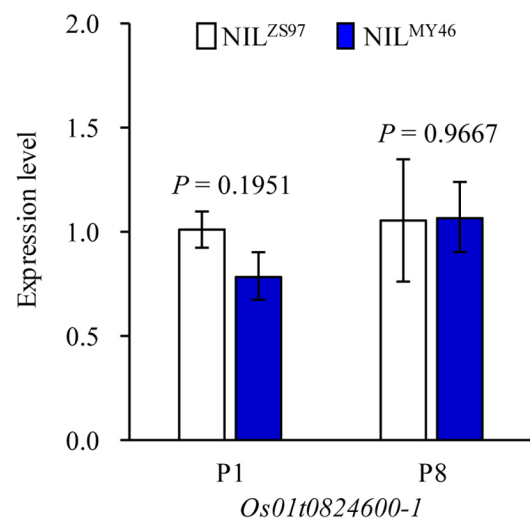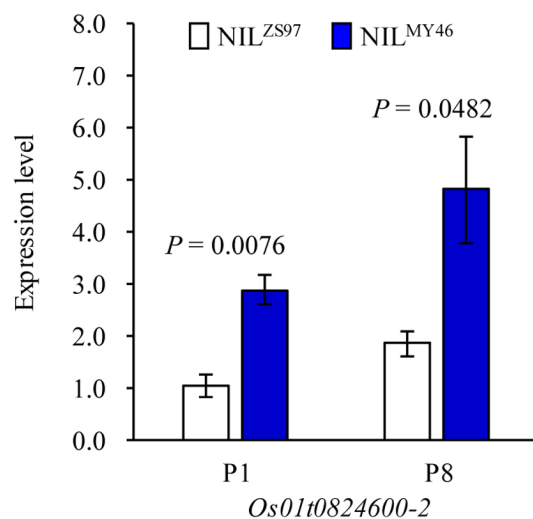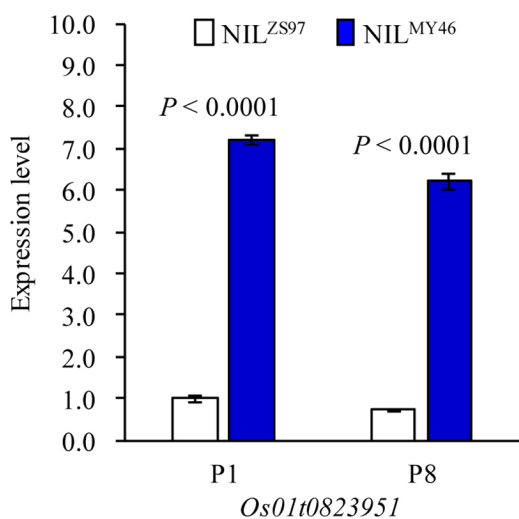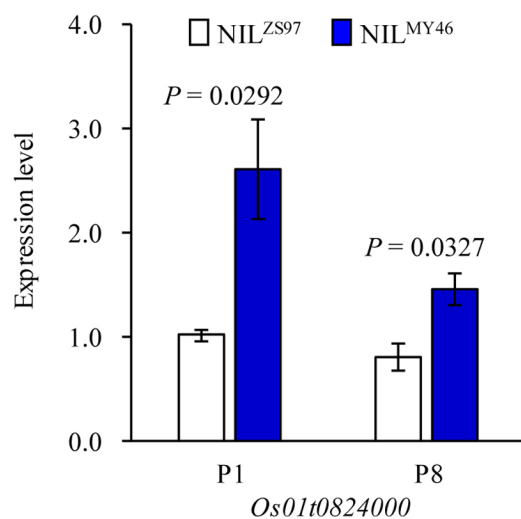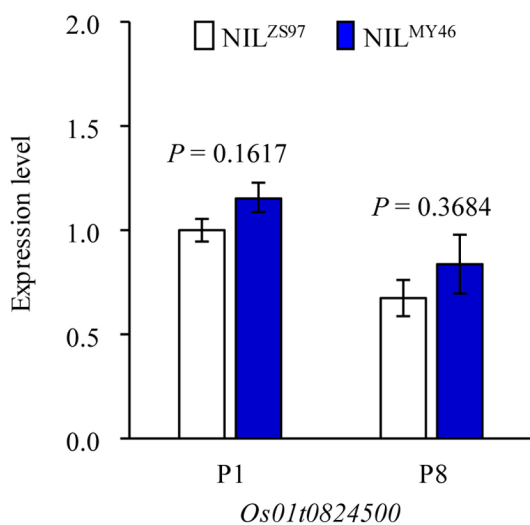

Supplement: Supplementary file 5 — Figure S4. Transcript levels of annotated genes in the qGS1-35.2 region. The experiment was performed using panicles of 1 cm (P1) and 8 cm (P8) collected from NILZS97 and NILMY46 for qGS1-35.2. The expression levels were normalized to Actin1 and related to P1 of NILZS97. Data are presented in mean ± s.e.m. (n = 3). A Student’s t-test was used to generate the P values. (PDF 2579 kb) [file 12284_2018_236_MOESM5_ESM.pdf]
